# Characterization of a quorum-sensing communication based on gamma-butyrolactones in *Rhodococcus erythropolis*

**DOI:** 10.3389/fmicb.2026.1849643

**Published:** 2026-07-27

**Authors:** Héloïse Bizière-Maco, Nathan Jordier, J. Francisco Barbosa-de-Bessa, Quentin Blancart Remaury, Fabien Perrier, Florie Desriac, Charly Alex Dupont, Sylvie Chevalier, Jack A. Connolly, Eriko Takano, Annabelle Merieau, Julien Verdon, Xavier Latour, Corinne Barbey

**Affiliations:** 1 Laboratory of Bacterial Communication and Anti-Infectious Strategies (CBSA UR4312, Formerly LMSM EA4312), University Rouen Normandie, University Caen Normandie, Normandie University, Rouen, France; 2 International Research Federation NOR-SEVE, University of Sherbrooke, Sherbrooke, QC, Canada; 3 Laboratory of Ecology and Biology of Interactions, UMR CNRS 7267, University of Poitiers, Poitiers, France; 4 UMR CNRS 7285, Institute of Environmental and Material Chemistry of Poitiers (IC2MP), University of Poitiers, Team Certified by the League Against Cancer, Poitiers, France; 5 Manchester Institute of Biotechnology, Department of Chemistry, School of Nature Sciences, Faculty of Science and Engineering, University of Manchester, Manchester, United Kingdom; 6 Biocontrol and Biostimulation for Agroecology Association (ABBA), Paris, France

**Keywords:** butyrolactones, communication, quorum-sensing, *Rhodococcus*, signals

## Abstract

Quorum sensing (QS) enables bacteria to coordinate collective behaviors, including the production of secondary metabolites with potential biotechnological applications, through the synthesis and detection of small signaling molecules. In *Streptomyces*, QS is well-described and mainly mediated by 2,3-disubstituted *γ*-butyrolactones (GBLs), which play key roles in the regulation of secondary metabolism and spore production. Despite their importance, GBL-based QS systems remain poorly characterized in other actinomycetal genera. Here, we investigated the distribution, structure, and function of GBL biosynthetic and regulatory systems within the genus *Rhodococcus*, focusing on the biocontrol strain *Rhodococcus erythropolis* R138. Comparative genomic analyses revealed that the *scbA* and *scbR* homologs, which are involved in GBL biosynthesis and detection, respectively, are widely conserved among *Rhodococcus* species and are organized within a conserved GBL gene cluster. Structural modeling using AlphaFold showed a high degree of conservation between ScbA and ScbR from *Streptomyces coelicolor* and their homologs in *R. erythropolis* R138. By combining liquid chromatography–mass spectrometry analyses with a GBL-specific reporter assay, we demonstrated that *R. erythropolis* R138 produces biologically active GBL(−like) molecules. Production of the investigated GBL molecules required the *scbA* gene, which restored spore production and promoted colony development in the *scbA*-deleted *S. coelicolor* strain during interaction with *R. erythropolis* R138. Transcriptional analyses further showed that both ScbR and a LuxR-like regulator may contribute to the fine-tuned regulation of *scbA* expression, revealing a complex regulatory network controlling GBL biosynthesis. This study provides novel and unexpected insights into the involvement of a LuxR homolog in regulating a QS system in Gram-positive bacteria. Together, these results demonstrate that functional GBL-based QS systems are conserved and active in *R. erythropolis* and likely widespread in the genus. This study expands current knowledge of QS in *Actinomycetota* and highlights the potential role of GBL signaling in regulating biotechnologically and ecologically relevant traits in *Rhodococcus*.

## 1 Introduction

Bacteria engage in cell-to-cell communication in particular by producing, releasing and detecting small diffusible or volatile signaling molecules (Barbey and Latour, 2024). A canonical form of this chemical communication, called quorum-sensing (QS), allows individual bacteria to monitor the size of their population and their spatial distribution by sensing the concentration of extracellular signaling molecules surrounding bacteria. When the population density threshold (i.e., the quorum) is reached, bacteria can then coordinate and accomplish cooperative behaviors that are energetically too costly for individual cells. This cell density-dependent mechanism ensures the correct timing and synchronization of physiological response to sudden environmental changes (Mukherjee and Bassler, 2019). QS systems are widespread among bacteria and consist of a signal synthase and a specific receptor, cytoplasmic transcription factor or transmembrane two-component histidine sensor kinases. Once formed, signal-receptor complexes direct the expression of genes involved among others in the production of public goods, often including the signal molecules themselves (autoinducers), and secondary metabolites such as antimicrobial compounds, extracellular enzymes, surfactants, and exopolysaccharides usable for biotechnological applications (Heilmann et al., 2015). Exploration, stimulation, or silencing of QS systems holds significant potential for progress in medical, biotechnological, and ecological research (Grandclément et al., 2016; Hartmann et al., 2024; Papenfort and Bassler, 2016).

The most studied and best-documented QS system is based on the production and detection of *N*-acyl homoserine lactones (AHLs) by Gram-negative bacteria (Papenfort and Bassler, 2016). These autoinducers are synthesized by LuxI-like synthases and sensed by LuxR-like specific receptors that generally activate transcription of their target genes once the autoinducer-LuxR complex is formed (Mukherjee and Bassler, 2019). In contrast, Gram-positive bacteria predominantly communicate with small secreted post-translationally processed peptides (Bhatt, 2018). A notable exception is bacteria belonging to the *Actinomycetota* phylum (formerly *Actinobacteria*) that mainly produce and detect 2,3-disubstituted *γ*-butyrolactones (GBLs) as signal molecules. To date, 29 different GBL structures have been identified (Creamer et al., 2021; Kudo et al., 2025). They all share the intrinsic 2,3-disubstituted γ-butyrolactone skeleton but differ in the length, branching and stereochemistry of their aliphatic side chains (Takano, 2006). When fully saturated, GBLs are also classified under the generic term butanolide. The first GBL molecule to be discovered is the A-factor of *Streptomyces griseus* (Khokhlov et al., 1967), and the others are studied almost exclusively in this bacterial genus, although their putative biosynthetic genes also appear to be present in other *Actinomycetes* genera (Creamer et al., 2021; Polkade et al., 2016). They act in nanomolar concentrations by binding to their cytoplasmic receptor protein usually described as transcriptional repressors belonging to the TetR family. Upon GBL binding, these transcriptional regulators dissociate from the promoter of the target genes involved in secondary metabolism including antibiotic production and the complex morphological differentiation of streptomycetes resulting in spore formation (Takano, 2006). The model organism of the genus *Streptomyces* is the most genetically described *Streptomyces coelicolor* A3(2; Bentley et al., 2002). This strain produces three *S. coelicolor* butanolides (named SCB1-3; Hsiao et al., 2009; Takano et al., 2000) through the activity of ScbA (*S. coelicolor* butanolide A synthase), the SCB key biosynthetic enzyme since deletion of the corresponding gene results in the inability to produce GBLs (Takano et al., 2001). SCBs are detected by the transcriptional regulator ScbR that represses its own gene expression and regulates *scbA* expression in a growth phase-dependent manner (Martin Sanchez, 2016; Takano et al., 2001). In the early stage of growth, ScbR represses *scbA* transcription and induces its expression in transition phase probably aided by another protein. SCBs molecules regulate directly the coelimycin production (Takano et al., 2005; Takano, 2006) and indirectly affect the production of two other antibiotics, actinorhodin and undecylprodigiosin (Takano et al., 2000; Takano et al., 2001).

Despite their significant role in the production of secondary metabolites with medical, ecological, and biotechnological relevance, the GBL-based QS systems are largely under-explored in other genera from *Actinomycetota* (Kudo et al., 2025; Polkade et al., 2016; Zhang et al., 2016). In the *Rhodococcus* genus, GBLs were only detected and characterized in *Rhodococcus jostii* RHA1 (Ceniceros et al., 2017a), and most recently in *Rhodococcus rhodnii* and *Rhodococcus rhodochrous* (Kudo et al., 2025). In our study, we showed that the *scbA* GBL synthase and *scbR* GBL receptor homologs were largely distributed among sequenced rhodococcal bacteria. We focused particularly on the *Rhodococcus erythropolis* species, because water and soil isolates belonging to this species are known to be excellent antibiotic producers, pollutant degraders, or plant protectors (de Carvalho and da Fonseca, 2005; Cirou et al., 2011, 2012; Ivshina et al., 2021). Thus, they play a crucial role in bioremediation, biocontrol, anti-biofouling, and anti-biofilm treatments (Afordoanyi et al., 2024; Bourigault et al., 2021; Chane et al., 2019; Kim et al., 2015; Ivshina et al., 2021). Among biocontrol agents, *Rhodococcus erythropolis* R138 is well-documented to protect plants against soft-rot phytopathogens (Barbey et al., 2018; Chane et al., 2019).

In this study, the GBL production by *R. erythropolis* R138 was characterized using liquid chromatography–mass spectrometry (LC–MS) analyses and a GBL-specific reporter assay. We demonstrated the essential role of *scbA* in GBL production by studying a *scbA* deletion mutant, and we discuss the potential contributions of additional genes within the GBL cluster defined in this study to GBL biosynthesis. We investigated how ScbR and a LuxR-like transcriptional regulator may contribute to the fine-tuned regulation of GBL biosynthesis, thereby ensuring the timely activation of QS target gene expression.

## 2 Materials and methods

### 2.1 Bioinformatic analysis of GBL gene cluster from *Rhodococcus* and *Streptomyces* strains

All complete genome sequences of *Rhodococcus* and *Streptomyces* strains were obtained from NCBI (National Center for Biotechnology Information).^1^ GBL gene clusters were analyzed using the NCBI Blast server and the clone manager software (professional edition version 9.00). To detect conserved functional domains in ScbR and ScbA, the amino acid sequences were aligned using the ClustalW algorithm. For phylogenetic tree construction, the conserved AfsA domains were identified using InterPro (EBI). The retrieved amino acid sequences were aligned with the MAFFT multiple alignment tool (Katoh and Standley, 2013) and subsequently curated using BMGE available through the NGPhylogeny platform (Criscuolo and Gribaldo, 2010). A maximum likelihood (ML) tree was inferred with FastME (Lefort et al., 2015), using the GTR evolutionary model, empirical equilibrium frequencies, SPR tree topology search, and approximate Bayes branch support as the statistical test (Guindon et al., 2010; Lemoine et al., 2018). The resulting phylogenetic ML Newick trees were edited using Newick Utilities (Junier and Zdobnov, 2010). All analyses were performed with NGPhylogeny.fr server (Lemoine et al., 2019). Graphic modifications were performed using the iTOL platform.^2^

### 2.2 Structural prediction of *Rhodococcus erythropolis* ScbA and ScbR proteins

Protein structure predictions for ScbA and ScbR were based on AlphaFold models (Jumper et al., 2021) available from the AlphaFold Protein Structure Database hosted by EMBL-EBI (Varadi et al., 2022). The 3D superposition of the protein structures was performed using PyMOL (PyMOL Molecular Graphics System, Version 2.0; Schrödinger, LLC).

### 2.3 Bacterial strains, growth and culture conditions

The characteristics of bacterial strains and plasmids are presented in the Supplementary Table S1. *R. erythropolis* strains were cultivated in liquid medium LBP containing 1% (w/v) Bacto-peptone (BD Difco, Le Pont de Claix, France), 0.5% (w/v) yeast extract (AES Chemunex, Bruz, France) and 1% (w/v) NaCl (Sigma Aldrich, St. Quentin Fallavier, France; Barbey et al., 2013) for conjugative transfer of DNA and transcriptional fusion analysis, in Supplemented Minimal Medium Solid SMMS medium (Ceniceros et al., 2017a) for GBL extractions, and in Minimum Salt Medium (MSM; Ceniceros et al., 2017a) for bacterial interaction assays. The Soya Flour Mannitol medium (SFM**)** containing 20 g/L soya flour (Sigma Aldrich, St. Quentin Fallavier, France), 20 g/L mannitol (Sigma Aldrich, St. Quentin Fallavier, France), and 20 g/L agar (AES Chemunex, Bruz, France) was used to grow *Streptomyces* strains for preparing spore suspensions. After incubation for 5 days, spores were stored in a 20% glycerol solution at −20 °C. For the GBL-specific reporter assay, the *S. coelicolor* LW94 pTE134 reporter strain was cultivated in DNAgar (BD Difco Nutrient Agar), as previously described (Biarnes-Carrera et al., 2018a). Luria-Bertani (LB) medium (AES Chemunex, Bruz, France) was used for the cultivation of *Escherichia coli* strains. When necessary, growth media were supplemented with kanamycin at a concentration of 5 μg/mL, 50 μg/mL and 400 μg/mL for *S. coelicolor, E. coli*, and *R. erythropolis*, respectively. Growth media were solidified with 15 g/L agar. All strains were grown on a rotary shaker (180 rpm) at 25 or 30 °C for *R. erythropolis* strains, at 30 °C for *S. coelicolor* strains, and at 37 °C for *Escherichia coli*.

### 2.4 *Rhodococcus erythropolis* and *S. coelicolor* interaction assays

The extemporaneously prepared MSM medium was used to analyze interactions between *R. erythropolis* R138 and *S. coelicolor* on agar plates. This medium was inoculated by applying 2 μL of *R. erythropolis* cultures with an optical density (OD) at 580 nm of 1.5. After 6 days of incubation at 30 °C, 2.5 × 10^6^ spores of *S. coelicolor* were spotted next to the *R. erythropolis* inocula. After a renewed incubation at 30 °C, spore formation was monitored after 1 day and at 24-h intervals thereafter, based on the appearance of the characteristic white pigmentation of aerial hyphae in *S. coelicolor*. Experiments were performed with three independent culture conditions for each strain.

### 2.5 GBL extraction, purification, and identification by liquid chromatography-mass spectrometry

*R. erythropolis* was grown in liquid Supplemented Minimum Medium (SMM) and then used to inoculate 80 SMMS plates per strain, standardized to an OD_580_ of 1.5. After 13 days of incubation at 30 °C, when the strains reached the secondary metabolic growth phase, GBLs were extracted using the ethyl acetate extraction method described by Hsiao et al. (2009). The 80 inoculated SMMS plates were cut into pieces and soaked in ethyl acetate with agitation for 2 h. The ethyl acetate was then recovered and evaporated under a chemical fume hood. The resulting organic phase, containing the extracted molecules, was resuspended in 500 μL of methanol. Ethyl acetate extracts were purified by solid-phase extraction (SPE) on C18 cartridges, using a methanol gradient for elution. Each fraction was evaporated to dryness by lyophilization. The same protocol was used to extract GBL from *S. coelicolor* M145, which served as a positive control.

To detect GBL molecules produced by *R. erythropolis* R138 strains, LC–MS/MS analyses were performed on an UHPLC Vanquish Flex system (Thermo Fisher Scientific, Germering, Germany) coupled to an Orbitrap Exploris 240 mass spectrometer (Thermo Fisher Scientific, Bremen, Germany) equipped with a Heated Electrospray Ionization (HESI) source, operating in negative ion mode. A reversed-phase C18 column (Luna Omega Polar C18, 100 × 2.1 mm, 1.6 μm, Phenomenex) was used for separation. The mobile phase consisted of a gradient of acetonitrile (Optima™ LC/MS Grade; Fisher Chemical™, Fisher Scientific, France) and water (Optima™ LC/MS Grade; Fisher Chemical™, Fisher Scientific, France), applied as follows: 2 mins at 2% acetonitrile, followed by a linear gradient from 2 to 80% acetonitrile over 23 min (2–25 min), remaining there for 1 min and then returning to the initial conditions within 1 min for a re-equilibration step during the last minute. The ionization parameters (HESI source) were set as follows: spray voltage 4.0 kV, capillary temperature 350 °C, and heater temperature 400 °C. Sheath, auxiliary and sweep gases were adjusted to 40, 20, and 0 arbitrary units, respectively. Analysis was done in negative mode with a resolution of 60,000 for Full MS scan over a mass range of 113–1,500 m/z, fixing the automatic gain control (AGC) with a custom value of 100% and a maximum injection time of 60 ms. Regarding the data-dependent MS^2^ scan, the properties were the following: 2 m/z of isolation window and a stepped normalized collision energy (NCE) of 30, 50%. A targeted mass list with the theoretical masses of the target compounds (m/z 241.1445, 255.1602, 242.1523 and 256.1680) was implemented with 5 ppm of tolerance. Data acquisition was performed with Xcalibur software (Thermo Fisher Scientific, USA) and was processed using FreeStyle (Thermo Fisher Scientific, USA).

### 2.6 Detection of GBL using the GBL-specific reporter assay

The GBL-specific reporter assays were performed following the previously described protocol (Biarnes-Carrera et al., 2018a). A suspension containing 2.5 × 10^6^ spores of *S. coelicolor* LW94 pTE134 was spread onto DNAgar plates supplemented with kanamycin (5 μg/mL). After the plates had dried, 10 μL of methanol extract was spotted onto the DNAgar plates, which were then incubated at 30 °C for 5 days. An ethyl acetate extract of *S. coelicolor* M145, resuspended in methanol, served as a positive control. Dried ethyl acetate resuspended in methanol was used as a negative control. These assays were performed with three biological replicates.

### 2.7 Construction of a *scbA* deletion mutant of *Rhodococcus erythropolis* R138, a *∆scbA-scbA* revertant R138 strain, and transcriptional fusion R138 strains

A markerless *scbA* deletion mutant was constructed as previously described (Barbey et al., 2013) using the oligonucleotide pairs scbA_up_fw/scbA_up_rv and scbA_do_fw/ scbA_do_rv (Supplementary Table S2) for the amplification of the *scbA* upstream and downstream regions (1,003 and 1,031 bp, respectively). The in-frame *scbA* deletion mutant containing a truncated version of the *scbA* gene (57 bp in length instead of 888 bp for the wild-type gene) was verified by PCR analysis and DNA sequencing. To generate the Δ*scbA*-*scbA* revertant strain, the *scbA* gene was amplified with the Q5 High Fidelity 2 × master mix (New England Biolabs), using primers scbA_up_fw and scbA_do_rv (Supplementary Table S2) and cloned as a 2,851-bp *Xba*I-*Bam*HI fragment into the conjugative vector pAKE604 (El-Sayed et al., 2001). After sequencing of the insert, the pAKE604-*scbA* plasmid was introduced into *R. erythropolis* R138 by biparental mating, as previously described (Barbey et al., 2013). The pEPR1 promoter probe vector (Knoppová et al., 2007) was modified to construct seven derivative vectors. The pEPR1-P*_scbA_:gfp*, pEPR1-*luxR*-P*_scbA_:gfp*, and pEPR1-P*_scbR_:gfp* plasmids were constructed by using the PscbA_fw and PscbA_rv primers (Supplementary Table S2) to amplify a 361 bp *Xba*I-*Bam*HI fragment, the luxRPscbA_fw and PscbA_rv primers (Supplementary Table S2) to amplify an 1,187 bp *Xba*I-*Bam*HI fragment, and the PscbR_fw and PscbR_rv primers (Supplementary Table S2) to amplify a 289 bp *Xba*I-*Bam*HI fragment, respectively. The pEPR1- P*_scbA_:gfp-*P_45_*:luxR,* pEPR1-P*_scbA_:gfp-*P_45_*:scbR,* pEPR1-P*_scbA_:gfp-*P_45_*:scbR-*P_45_*:luxR,* pEPR1-P*_scbR_:gfp,* pEPR1-P*_scbR_:gfp-*P_45_*:scbR* plasmids were obtained by cloning the P_45_*:luxR* or P_45_*:scbR* fragment into the *Nsi*I or *Nhe*I pEPR1 restriction site, respectively. These P_45_-containing fragments were obtained by amplifying the P_45_ promoter from the pRAG5 plasmid with the P45_fw and P45_rv primers (Supplementary Table S2) and the corresponding *luxR* or *scbR* coding region with the lux_fw and lux_rv primers, scbR_fw and scbR_rv primers, respectively. Then, the P_45_ promoter and the corresponding coding region were ligated, and the resulting DNA fragments reamplified before their introduction into the pEPR1 plasmid. After sequencing, plasmids harboring transcriptional fusions were introduced into *R. erythropolis* R138 by electroporation, as previously described (Barbey et al., 2013).

### 2.8 Analysis of transcriptional fusion strains by confocal laser scanning microscopy (CLSM) and dot blot assays

Bacterial strains transformed with plasmids harboring transcriptional fusions were cultivated in LBP broth. Before CLSM, bacterial cells were immobilized in a 1.5% agar pad prepared with growth medium and covered with a coverslip. Images were acquired on a LEICA Stellaris 8 CLSM (Leica Microsystems, Wetzlar, Germany) using LAS X software, with identical gain and offset settings applied to all images. To excite the GFP in bacterial cells, a 488 nm laser with 509 nm emission filters was used. Three independent cultures were analyzed for each condition. To quantify GFP production, proteins were extracted from each rhodococcal culture by sonication, as previously described (Barbey et al., 2012), and subsequently examined by dot blot using anti-GFP (Rockland, Le Perray-en-Yvelines, France) and anti-RecA (Abcam, Cambridge, United Kingdom) antibodies. Prior to nitrocellulose membrane deposition, proteins were quantified using the Bradford method. A total of 20 μg of protein per condition was loaded onto two separate membranes. After drying, membranes were incubated for 1 h in blocking buffer. Then, the membranes were sequentially incubated for 1 h each in primary (anti-GFP or anti-RecA) and secondary (alkaline phosphatase-conjugated anti-rabbit IgG; Invitrogen) antibody solutions. The membranes were developed with the alkaline phosphatase (AP) colorimetric kit (Bio-Rad) according to the manufacturer’s instructions. Quantitative densitometry of anti-GFP signal normalized to anti-RecA signal was performed using ImageJ.

## 3 Results

### 3.1 *scbA* and *scbR* homologs are widespread among the *Rhodococcus* genus

**Figure 1:**
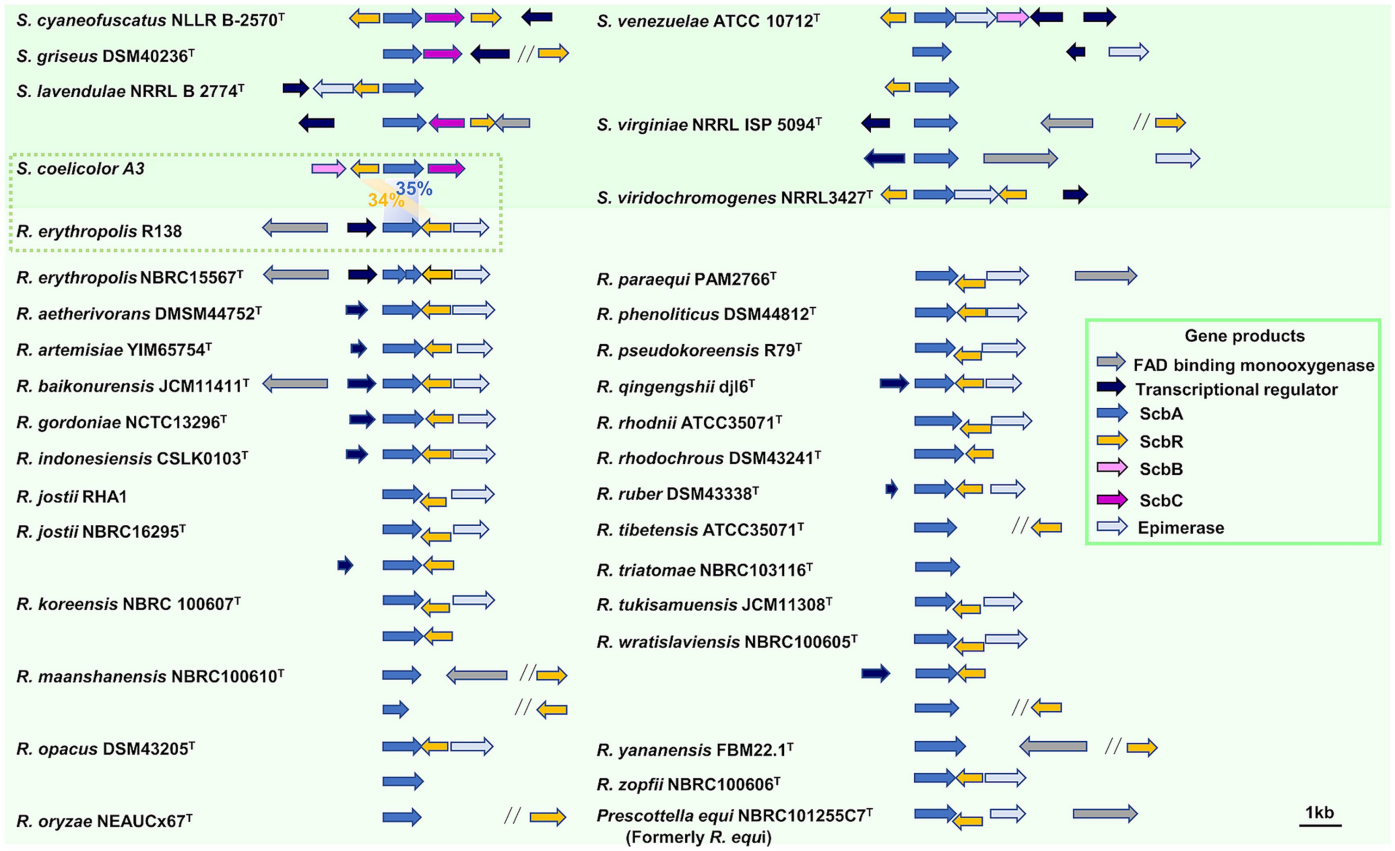
Comparative organization of the predicted gene clusters involved in γ-butyrolactone (GBL) synthesis and detection in *Rhodococcus* and *Streptomyces* species. The predicted γ-butyrolactone gene cluster arrangement from *Rhodococcus erythropolis* R138 was compared with the well-characterized corresponding cluster in the model species *S. coelicolor*, as well as with the γ-butyrolactone clusters predicted in type and some reference strains of *Streptomyces* and *Rhodococcus* species. Type strains are marked with a superscript T. Only *Streptomyces* species with experimentally characterized GBLs are indicated here. Homologous genes are represented in the same color and drawn to scale. A double slash is used to show that genes are spaced apart. Some *scbA* and *scbR* genes overlap at their 3′ ends. This overlap is represented when *scbR* is shifted relative to *scbA*. In the model species *S. coelicolor*, the *scbA*, *scbB*, and *scbC* genes encode enzymes for GBL synthesis while *scbR* encodes the GBL receptor protein. Only *scbR* homolog is detected in close proximity to the key *scbA* homolog in the genome of *R. erythropolis* R138. Sequence identities between each of the encoded proteins are indicated. An extended GBL gene cluster including additional genes encoding two enzymes, FAD-containing monooxygenase and epimerase, and a transcriptional regulator, was proposed based on its conserved presence across all sequenced *R. erythropolis* strains containing *scbA*.

The existence of a GBL gene cluster in *R. erythropolis* R138 was initially predicted based on prior *in silico* analyses (Ceniceros et al., 2017a; Latour et al., 2013). This cluster includes an *scbA* homolog encoding the key enzyme in GBL synthesis, an *scbR* homolog responsible for GBL detection and a gene encoding an NAD-epimerase/dehydratase (Figure 1). This *in silico* analysis was extended in the present study by screening all annotated genomes of *R. erythropolis* for the presence of the GBL gene cluster using the NCBI databases and NCBI BLAST server. The results revealed that all annotated genomes of *R. erythropolis* strains, carrying a *scbA* homolog, harbor a highly conserved gene cluster corresponding to an expanded GBL gene cluster, distinguished by the presence of two additional genes encoding a putative LuxR-like transcriptional regulator and a FAD-dependent monooxygenase. This GBL gene cluster is bordered by genes that are not conserved between *R. erythropolis* strains (Supplementary Table S3). Protein sequence comparison revealed that *R. erythropolis* R138 ScbA and ScbR share 35 and 34% identity, respectively, with their *S. coelicolor* homologs (Supplementary Figures S1, S2). *R. erythropolis* R138 ScbA contains the two typical conserved AfsA repeats (Pfam03756 conserved domain; Supplementary Figure S1). The two additional genes, *scbB* and *scbC*, implicated in GBL biosynthesis in *S. coelicolor*, were not found in the immediate vicinity of the *scbA* gene in the *R. erythropolis* genome R138.

The presence of the proposed GBL gene cluster was investigated in the genomes of type strains of the *Rhodococcus* genus (Figure 1). All analyzed *Rhodococcus* strains harbor the *scbA* and *scbR* genes, with *scbA* in most strains flanked by *scbR* in a convergent orientation. Most strains carry a single copy of *scbA*, whereas *Rhodococcus wratislaviensis* harbors three copies. In the *R. erythropolis* NBRC15567^T^ type strain, the *scbA* gene is organized in two open reading frames with a partial deletion. The epimerase-encoding gene is found in the majority of strains. A transcriptional regulator encoding gene is located upstream of *scbA*, except in GBL clusters where the *scbA* and *scb*R genes overlap at their 3′ ends. The monooxygenase-encoding gene was detected in approximately 25% of the analyzed strains. Analysis of the GBL cluster in the genomes of *Streptomyces* strains with experimentally characterized GBLs (Creamer et al., 2021; Kudo et al., 2025) revealed the presence of *scbA* and *scbR* homologs, typically arranged in a divergent orientation, and transcriptional regulator genes with various positions in the clusters. The epimerase encoding gene was detected in all analyzed *Streptomyces* strains except for *Streptomyces cyaneofuscatus*, *S. coelicolor*, and *S. griseus* whereas the monooxygenase encoding gene was only found in the *Streptomyces lavendulae* and *Streptomyces virginiae* strains. This comparative analysis of GBL clusters indicated a conserved organization in *Rhodococcus* strains, whereas *Streptomyces* strains displayed a markedly more diverse set of GBL clusters.

**Figure 2:**
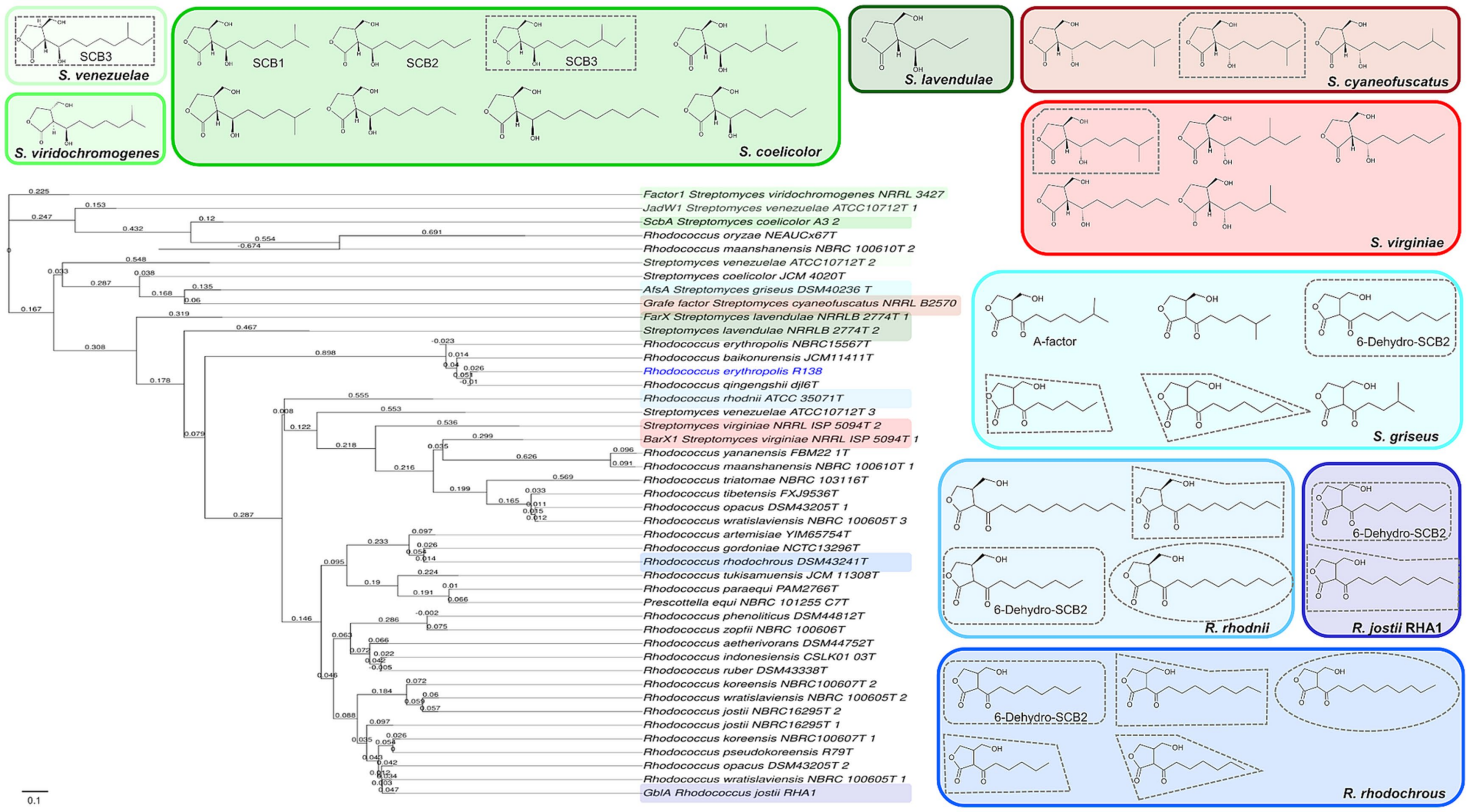
Phylogenetic analyses of ScbA homologs from *Rhodococcus* and *Streptomyces* strains in relation to the chemical structures of their corresponding GBLs. The phylogenetic tree was constructed based on the conserved AfsA domains (Pfam03756). Sequence alignment was performed with MAFFT and the phylogenetic tree was generated using the NGPhylogeny platform. A maximum likelihood tree was inferred with FastME using the GTR model and approximate Bayes branch support. ScbA of *R. erythropolis* R138 is highlighted in blue. Scale bar represents the mean number of amino acid substitutions per site. GBL compounds experimentally linked to their respective *scbA* gene are mapped to the tree. Colored boxes delineate the three main groups of GBL: A-factor-type GBLs (in blue), which contains a keto group in the carbon 6 of the molecule; IM-2-type GBLs (in green), with a hydroxyl group in the same carbon in the (R) configuration; and VB-type GBLs (in red), also with a hydroxyl group in the same carbon but in an (S) configuration. Bracketed numbers are used to refer to the number of putative *scbA* genes identified in a single bacterial strain. GBL compounds observed in several species are indicated by dashed lines.

To investigate the evolutionary relationships among ScbA homologs from *Rhodococcus* type strains and the experimentally characterized *Streptomyces* ScbA proteins, a phylogenetic tree was constructed based on the two conserved AfsA domains corresponding to the putative active site of ScbA. The phylogenetic tree revealed two main clusters: one predominantly composed of *Streptomyces* ScbA homologs and another mainly comprising *Rhodococcus* sequences, with some exceptions where *Rhodococcus* sequences clustered within the *Streptomyces* group and vice versa (Figure 2). The *Streptomyces* cluster formed a homogeneous group with short branch lengths, indicative of strong conservation within the AfsA domains. In contrast, the *Rhodococcus* cluster displayed greater variability and longer branch lengths, with numerous internal nodes, reflecting high diversity among AfsA domains and consequently lower conservation. Within the *Rhodococcus* genus, GBLs have so far been characterized in only three *Rhodococcus* species: *R. jostii* RHA1, *R. rhodnii*, and *R. rhodochrous* (Figure 2). All GBLs produced by the three *Rhodococcus* strains belong to the A-factor–type GBLs, whereas *Streptomyces* strains produce GBLs from three GBL groups: A-factor- (first described in *S. griseus*), IM-2- (initially identified in *S. lavendulae*), and VB- (first reported in *S. virginiae*) type GBLs. The ScbA homologs from the three *Rhodococcus* species and *R. erythropolis* R138 displayed phylogenetic divergences relative to the *S. coelicolor* A3(2) model strain, as indicated by cumulative evolutionary distances of 1.439, 1.582, 1.561, and 1.746, respectively. In contrast, they showed a closer phylogenetic relationship to the *S. virginiae* ScbA homolog BarX1, with cumulative evolutionary distances of 0.707, 0.85, 0.829, and 1.015, respectively. This phylogenetic proximity suggests that *Rhodococcus* strains may produce GBLs with structural similarities to those synthesized by *S. virginiae*. However, the GBLs produced by the three characterized *Rhodococcus* strains differ from those synthesized by *S. virginiae* strains.

### 3.2 ScbA and ScbR from *Rhodococcus erythropolis* R138 and *S. coelicolor* share highly similar predicted 3D structures

To date, no crystal structure has been reported for ScbA from *S. coelicolor* or any ScbA homologs. A structural model of ScbA was proposed based on its homology to the crystallized fatty acid synthases FabA and FabZ. Indeed, the regions around the active sites of FabA and FabZ show significant similarity to the typical AfsA repeats of ScbA (Hsiao et al., 2007). In the present study, an updated version of the ScbA 3D structural model was generated using the AlphaFold structure prediction algorithm (Jumper et al., 2021). The ScbA 3D structural models from *R. erythropolis* and *S. coelicolor* were compared by superimposing them in PyMOL (Supplementary Video 1). The ScbA model from *R. erythropolis* showed a close structural similarity to that of *S. coelicolor*, with a Root Mean Square Deviation (RMSD) of 1.137 Å (for 1395/1743 atoms), indicating a high degree of conservation. The key amino acid residues of *S. coelicolor* ScbA, namely E78, R81, E240, and R243 within the putative active site, and R174 in the loop connecting the two AfsA repeats, previously identified through point mutagenesis (Hsiao et al., 2009; Martin Sanchez, 2016), were found to be conserved in the *R. erythropolis* R138 sequence (Supplementary Video 1).

ScbR is the GBL receptor that functions as a transcriptional regulator by binding to the promoters of target genes. To date, the only crystallized ScbR homologs are CprB from *S. coelicolor* (Bhukya et al., 2014; Natsume et al., 2004) and TylP from *Streptomyces fradiae* (Mohd-Sharif et al., 2017). The GBL binding to CprB and TylP has not been experimentally demonstrated, hence their designation as pseudoreceptors. The availability of these structures makes it possible to predict the 3D structure of ScbR from *R. erythropolis* R138 and *S. coelicolor*. Alignment of the two structures showed that the two ScbR proteins were highly conserved, with a RMSD of 1.189 Å (for 1028/1207 atoms; Supplementary Video 2). Analysis of the ScbR 3D structure highlighted the two distinct functional domains: the N-terminal DNA binding domain featuring a helix-turn-helix motif and the C-terminal regulatory ligand binding domain. The latter domain contains a large cavity which seems to be a ligand-binding pocket surrounded by helices α4, α5, α6, α7, and α8. Amino acids critical for DNA binding (Val-41, Pro-115; Onaka and Horinouchi, 1997) and ligand interaction (Trp-127; Sugiyama et al., 1998) were conserved in the *R. erythropolis* ScbR sequence (Supplementary Video 2; Supplementary Figure S2).

### 3.3 *Rhodococcus erythropolis* R138 triggers spore production in the *scbA*-deleted *S. coelicolor* strain upon interaction

**Figure 3:**
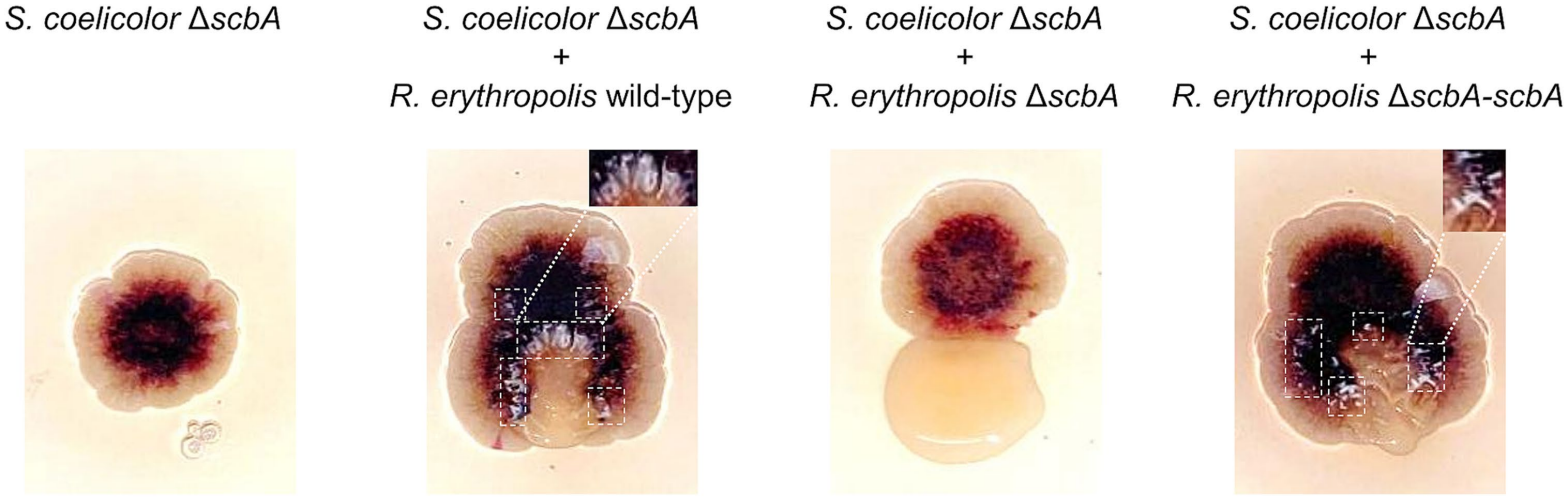
Interactions between the *R. erythropolis* R138 wild-type, Δ*scbA*, and Δ*scbA-scbA* strains with the *S. coelicolor* mutant strain lacking *scbA* on MSM agar. *R. erythropolis* R138 strains were spotted and incubated for 6 days before spores of *S. coelicolor* LW94 (Δ*scbA*) were inoculated adjacent to the *Rhodococcus* colonies. Images were acquired after a further 7 days of incubation. The white pigmentation characteristic to spore production is indicated by dashed white box. A close-up of the white mycelium is shown at the top of the image. The pictures shown are representative of three independent experiments.

The interaction of *R. erythropolis* R138 with the *S. coelicolor* LW94 mutant lacking *scbA* was assessed by spotting the two strains next to each other on an agar plate. The wild-type, *scbA* deleted mutant (Δ*scbA*), and revertant (Δ*scbA*-*scbA*) strains of *R. erythropolis* R138 were cultured separately on MSM plates for 4 days, after which *S. coelicolor* LW94 was inoculated next to the rhodococcal strain. A control plate was prepared by culturing *S. coelicolor* LW94 in the absence of any rhodococcal strain. After an additional 7 days of incubation, colony development of *S. coelicolor* LW94 was enhanced in the presence of the *R. erythropolis* R138 wild-type and Δ*scbA*-*scbA* strains compared with the Δ*scbA* mutant. Notably, the *S. coelicolor* colony formed around the *R. erythropolis* wild-type and Δ*scbA*-*scbA* strains, suggesting a possible attraction to these rhodococcal strains. This phenomenon was not observed in the presence of Δ*scbA*. Furthermore, *S. coelicolor* LW94 produced a powdery aerial mycelium in the presence of the rhodococcal wild-type and *ΔscbA*-*scbA* strains, exhibiting the characteristic white pigmentation of aerial hyphae differentiating into spore chains (Figure 3; Vargas-Gómez et al., 2025). This white pigmentation first appeared in the immediate proximity to the rhodococcal inoculum. By contrast, no such development occurred when *S. coelicolor* LW94 was cultured alone or beside Δ*scbA*. Since *S. coelicolor* LW94 lacks *scbA*, required for GBL synthesis, the results support the hypothesis that GBLs produced by *R. erythropolis* R138 wild-type, and Δ*scbA*-*scbA* strains may induce induce spore formation, consistent with the known role of GBLs in coordinating sporulation in *Streptomyces* species (Takano, 2006).

### 3.4 *Rhodococcus erythropolis* R138 produces GBL(−like) molecules

**Figure 4:**
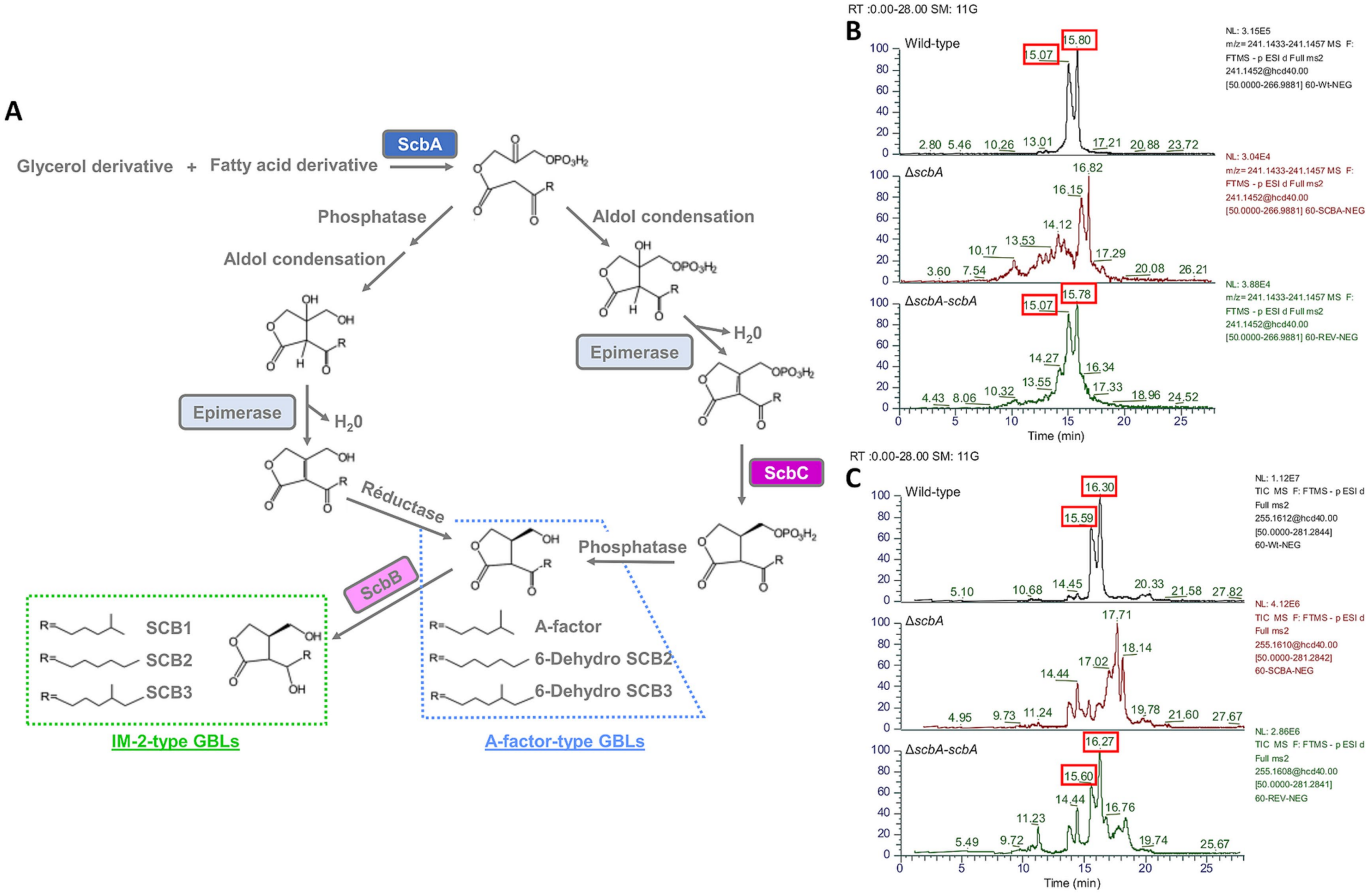
Production of GLB(−like) molecules by *R. erythropolis* R138. **(A)** The proposed biosynthetic pathways for A-factor- and IM-2-type GBL biosynthesis in *Streptomyces* species (adapted from Ceniceros et al., 2017a). The ScbA, ScbB, ScbC, and Epimerase enzymes are shown at their putative catalytic steps. A-factor-type GBLs are characterized by the presence of a keto group at carbon 6 of the GBL backbone, a name derived from the first identified GBL molecule in *S. griseus*, known as A-factor. IM-2-type GBLs, including SCB (*Streptomyces coelicolor* Butyrolactones) molecules, are defined by the presence of a hydroxyl group at carbon 6 in the (R) configuration. **(B)** Extracted ion chromatograms (EIC) for m/z 241.145 with a ∆m = ± 5 ppm from the LC–MS/MS analysis of ethyl acetate extracts of the *R. erythropolis* R138 wild-type, Δ*scbA* and Δ*scbA*-*scbA* strains. The EIC for m/z 241.145 showed two peaks at 15.07 and 15.80 min present in the wild-type and Δ*scbA*-*scbA* strains, but not in the Δ*scbA* mutant. This exact mass matches the A-Factor or 6-dehydro-SCB2 mass, known to be an intermediate in the synthesis of the GBL SCB2 in *S. coelicolor*. **(C)** EIC for m/z 255.160 with a ∆m = ± 5 ppm from the LC–MS/MS analysis of ethyl acetate extracts of the *R. erythropolis* R138 wild-type, Δ*scbA* and Δ*scbA*-*scbA* strains. This showed two peaks at 15.59 and 16.30 min in the wild-type and Δ*scbA*-*scbA* strains, whereas no corresponding peaks were detected in the Δ*scbA* mutant. This mass may correspond to 6-dehydro-SCB3, known to be an intermediate in the synthesis of the GBL SCB3 in *S. coelicolor*.

The capacity of *R. erythropolis* R138 to produce GBL(−like) molecules was investigated by liquid chromatography coupled to a mass spectrometer (LC–MS/MS) and by using a GBL-specific reporter assay (Biarnes-Carrera et al., 2018a). The confirmation of GBL(−like) molecules was carried out by analyzing resuspensions of dried ethyl acetate extracts from solid cultures of the *R. erythropolis* R138 wild-type, Δ*scbA*, and Δ*scbA*-*scbA* strains. LC–MS/MS data acquired were analyzed searching for metabolites with masses corresponding to those of previously described GBL molecules (Biarnes-Carrera et al., 2018b; Ceniceros et al., 2017a; Joo et al., 2007; Kudo et al., 2025). The absence of authentic standards, which are commercially unavailable, hindered the precise identification of the specific molecule among the GBL molecule family. Based on the predicted biosynthetic pathway of GBLs in *Streptomyces* species (Figure 4A), *R. erythropolis* R138, whose genome lacks homologs of the *scbB* and *scbC* genes, is expected to produce A-factor-type GBLs including A-factor, 6-dehydro-SCB2, and/or 6-dehydro-SCB3. An extracted ion chromatogram (EIC) was generated to search for the corresponding masses (∆m ± 5 ppm) in the chromatogram. In the EIC (−) for m/z 241.145, which corresponds with the mass exact of A-Factor or 6-dehydro-SCB2, specifically for the adduct [M-H]^−^, two peaks at 15.07 and 15.80 min were detected in the analysis of wild-type and Δ*scbA*-*scbA* strains, but not in Δ*scbA* (Figure 4B). MS^2^ fragmentation of the m/z 241.145 precursor detected at 15.07 retention time for the wild-type strain (Supplementary Figure S3A), produced characteristic fragment ions at m/z 223.134 (H_2_O loss) and 197.155 (CO_2_ loss), from the GBL(−like) molecule. These fragments may match the fragmentation pathway reported in previous publications, involving ring opening through CO₂ loss and/or dehydrations and *β*-eliminations on the alkyl chain. The Δ*scbA*-*scbA* strain exhibited an essentially identical fragmentation pattern and relative intensity distribution. In contrast, the Δ*scbA* mutant strain displayed an altered MS^2^ spectrum with changes in relative fragment intensities and the absence or reduced abundance of fragments, with the major m/z ion being 241.181. Therefore, the distinct MS^2^ fragmentation profile as the absence of that elution peak in the mutant strain, strongly supported the role of the *scbA* gene in the biosynthesis of the detected GBL(−like) compound. Even more, the MS^2^ spectrum at 15.80 retention time in the wild-type strain was in concordance, as it was indicated by the presence of the same fragments with a similar proportion, for example, the following: 143.108, 115.040, and 85.029 and 167.144. These same fragments, generated by the fragmentation of the same precursor ion were also detected in the Δ*scbA*-*scbA* strain at a lower abundance but maintaining the relative abundance to m/z 241.145. Therefore, the data indicated that there might be at least two metabolites with the same molecular formula and very similar structures, based on their fragmentation patterns, which could correspond to structural isomers such as A-Factor and 6-dehydro-SCB2. Previous works have also reported this feature in the detection of these molecules (Biarnes-Carrera et al., 2018b; Ceniceros et al., 2017a; Joo et al., 2007; Takano et al., 2000).

Regarding the m/z 255.160 [M-H]^−^, which matches the theoretical value expected for 6-dehydro-SCB3 GBL molecules within ± 5 ppm mass error, in the corresponding EIC (−), two main peaks were detected at 15.60 and 16.30 min in the wild-type and Δ*scbA*-*scbA* extracts (Figure 4C). MS^2^ analysis of the m/z 255.160 precursor ion in the wild-type extract for 15.57 and 16.30 min peaks showed a major fragment ion m/z 167.144, together with other fragments m/z 211.134, 153.128, 125.097, and 83.050 (Supplementary Figure S3B). The Δ*scbA*-*scbA* strain exhibited an essentially identical fragmentation pattern and relative intensity distribution. This feature was markedly altered in the Δ*scbA* mutant strain, which exhibited a distinct MS^2^ fragmentation pattern at both retention times, suggesting the absence of the native GBL species. Taken together, these data supported the presence of a GBL(−like) molecule that may correspond to 6-dehydro-SCB3 in the wild-type and Δ*scbA*-*scbA* strains, and demonstrated that its production depended on the *scbA* gene.

GBL production by *R. erythropolis* R138 was further investigated using an alternative approach based on a *S. coelicolor* reporter strain impaired in GBL synthesis. This reporter strain harbors the plasmid pTE134, which carries a kanamycin resistance gene whose expression is repressed by the transcriptional regulator ScbR in the absence of GBLs. Reporter strain growth is permitted only in the presence of GBLs, which bind to ScbR and relieve repression, thereby allowing transcription of the kanamycin resistance gene (Biarnes-Carrera et al., 2018a). On kanamycin-supplemented agar plates, a halo of reporter strain growth was observed at the center of the plates in the presence of the *S. coelicolor* ethyl acetate extract, used as a positive control, as well as in the presence of the *R. erythropolis* R138 ethyl acetate extract, whereas no growth was observed under the negative control condition consisting of solvent application (Supplementary Figure S4). This bioassay exhibits high specificity, as alterations in the aliphatic side chain of GBLs markedly reduce their binding affinity to ScbR (Hsiao et al., 2009), and generic GBL molecules fail to trigger reporter strain growth (Bhukya et al., 2014). Due to the high specificity of this assay, the observed growth of the reporter strain in the presence of the *R. erythropolis* R138 ethyl acetate extract demonstrated that this strain produced GBL-like molecules that bound the *S. coelicolor* receptor ScbR, allowing transcription of the kanamycin resistance gene.

### 3.5 ScbR and LuxR appear to be involved in the regulation of *scbR* and *scbA* expression

**Figure 5:**
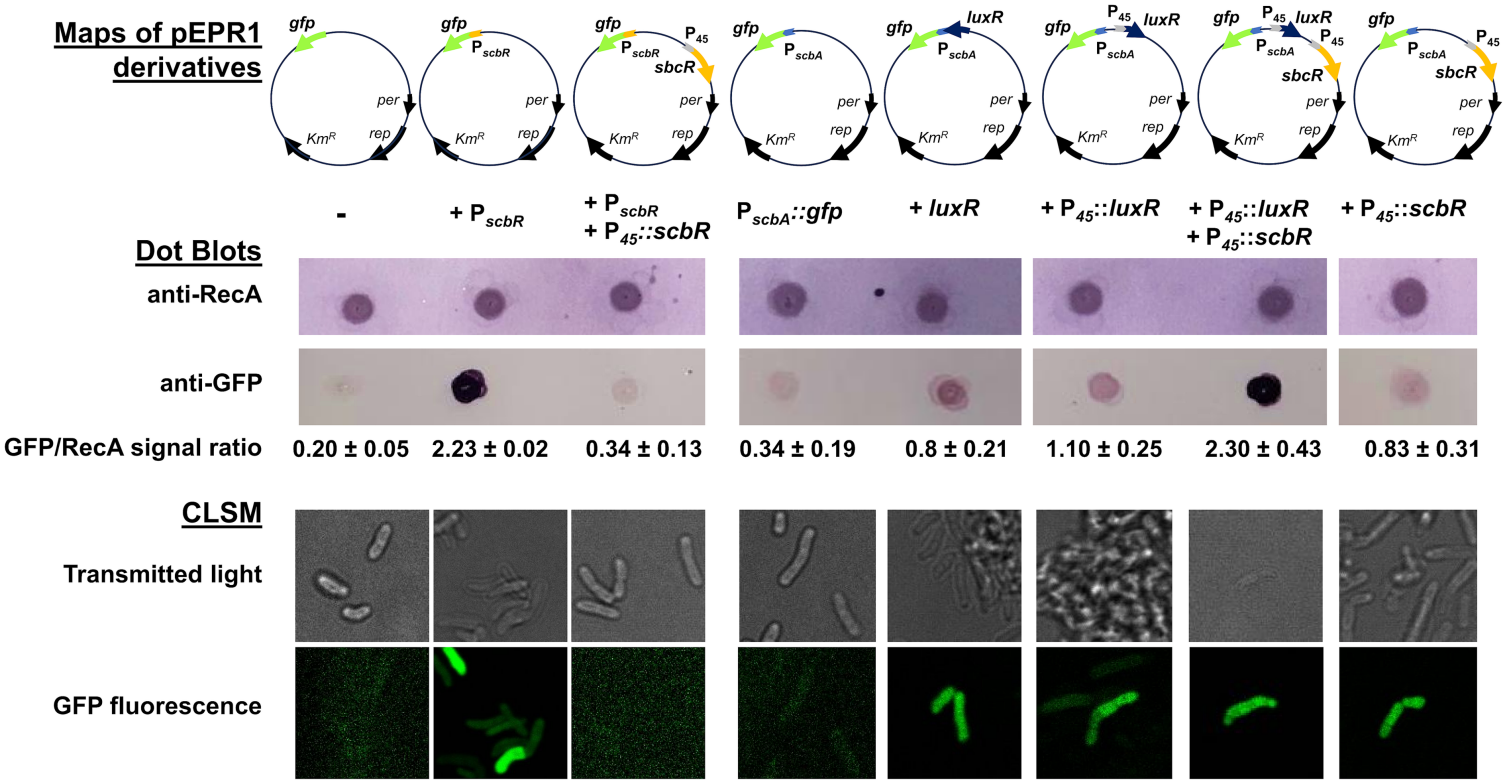
Promoter activity analysis of the *scbA* and *scbR* genes through measurement of GFP production and fluorescence in *R. erythropolis* R138 strains carrying transcriptional fusions with the *gfp* reporter gene. The promoterless pEPR1 vector, indicated as “-“under the plasmid map, was engineered to carry, upstream of the *gfp* gene, the *scbR* promoter (+P*_scbR_*), *scbA* promoter (+P*_scbA_*) or an extended *scbA* promoter region including *luxR* (+*luxR*P*_scbA_*). Additional constructs were generated by fusing the strong P_45_ promoter to the coding sequence of *scbR*, *scbA*, or *luxR*. These transcriptional fusions were designated +P_45_:*scbR*, +P_45_:*scbA*, and +P_45_:*luxR*. Maps of the resulting vectors are displayed. Dot blot assays using anti-GFP antibodies were conducted to quantify the GFP production by *R. erythropolis* R138 constructs. Protein extracts from all *R. erythropolis* R138 strains were spotted onto the same nitrocellulose membrane to ensure consistent detection conditions. A parallel dot blot with anti-RecA antibodies was carried out to confirm equal protein loading across conditions. The negative control consists of a protein extract from the *R. erythropolis* R138 strain carrying the promoterless pEPR1 vector. Fluorescence activity of the transcriptional fusion strains was analyzed by CLSM. Dot blot and CLSM images shown are representative of three independent experiments. The signal intensity of each spot was quantified using ImageJ. Anti-GFP signal was normalized to anti-RecA signal to determine the GFP/RecA signal ratio. Mean values and standard deviations are indicated below the dot blots and are also presented as histograms with statistical analyses in Supplementary Figure S5.

Transcriptional fusions were generated using the pEPR1 plasmid containing a promoterless *gfp* reporter gene to investigate the regulation of *scbR* and *scbA* expression. Fluorimetric quantification of GFP produced by *R. erythropolis* strains is hindered by the distinctive cell envelope and carotenoid production, which generate autofluorescence, increasing background and potentially masking GFP signals in whole-cell measurements (Cappelletti et al., 2020; Knoppová et al., 2007). An alternative approach is to extract proteins from the rhodococcal cells and quantify GFP levels with anti-GFP antibodies. Dot blot assays were performed by applying protein extracts from the *R. erythropolis* wild-type strains containing pEPR1 vector with or without transcriptional fusions onto the same nitrocellulose membrane to ensure uniform detection. Analysis of the dot blot using anti-RecA antibodies revealed uniform spot intensity for all *R. erythropolis* strains, confirming that equivalent amounts of protein were loaded for each extract (Figure 5). The intensity of the spot corresponding to the protein extract from *R. erythropolis* pEPR1 empty vector served as a threshold for GFP detection (Supplementary Figure S5). No fluorescence was detected for this negative control strain by CSLM (Figure 5). Introduction of the *scbR* promoter into the pEPR1 vector resulted in GFP production, as evidenced by the strong spot intensity which was 11-fold higher than the threshold (Supplementary Figure S5). The addition of the coding region of *scbR* fused to the strong P_45_ promoter into the pEPR1-P*_scbR_:gfp* vector abolished GFP production in the *R. erythropolis* strain pEPR1- P*_scbR_:gfp*-P_45_*:scbR*, indicating that expression of *scbR* is negatively regulated by ScbR (Figure 5). CSLM analysis confirmed the lack of GFP production by the *R. erythropolis* pEPR1-P*_scbR_:gfp*-P_45_*:scbR* strain, whereas the pEPR1-P*_scbR_:gfp* strain displayed GFP fluorescence (Figure 5). In contrast to *scbR*, insertion of the *scbA* promoter into the pEPR1 vector failed to induce GFP expression, as indicated by a spot intensity at the detection threshold (Supplementary Figure S5). No fluorescence was produced by the *R. erythropolis* pEPR1-P*_scbA_:gfp* strain (Figure 5). The insertion of an extended *scbA* promoter region containing the *luxR* gene into pEPR1 resulted in detectable GFP production by *R. erythropolis* pEPR1-*luxR*-P*_scbA_:gfp*, with a GFP signal 2.5-fold greater than that observed in the *R. erythropolis* R138 strain with pEPR1-P*_scbA_*:*gfp* (Supplementary Figure S5). This result correlated with the fluorescence observed under CSLM. This positive regulatory role of *luxR* on *scbA* expression was supported by GFP production and fluorescence detection in the *R. erythropolis* strain carrying the pEPR1-P*_scbA_:gfp* vector, in which the *luxR* coding region fused to the P_45_ promoter was inserted at a position distant from the *scbA* promoter. The resulting vector was designated pEPR1-P*_scbA_:gfp*-P_45_*:luxR*. The role of ScbR in the regulation of *scbA* was investigated by inserting the P_45_*:scbR* transcriptional fusion into the pEPR1-P*_scbA_:gfp* vector. *ScbR* expression led to the production of GFP and fluorescence by the *R. erythropolis* pEPR1-P*_scbA_:gfp*-P_45_*:scbR* strain, suggesting a positive regulatory role of ScbR in *scbA* expression. Dual expression of *scbR* and *luxR* by insertion of P_45_*:scbR* and P_45_*:luxR* resulted in strong GFP production, as evidenced by high spot intensity which was 7-fold higher than that detected in the *R. erythropolis* R138 strain with pEPR1-P*_scbA_*:*gfp* (Supplementary Figure S5).

## 4 Discussion

QS is used by bacteria to synchronize gene expression in response to population density, thereby modulating collective behaviors essential for survival and adaptation to specific ecological niches (Mukherjee and Bassler, 2019). These QS-regulated processes include the production of active secondary metabolites that hold medical, biotechnological, and ecological significance. Members of the *Rhodococcus* genus are renowned for their remarkable metabolic versatility, which provides significant potential in bioremediation, chemical biocatalysis, and natural product exploration (Cappelletti et al., 2020; Hu et al., 2024; Zampolli et al., 2022). These metabolic potential results, among other factors, from the large number of biosynthetic and biocatalytic gene clusters present in *Rhodococcus* genomes. Doroghazi and Metcalf (2013) demonstrated that the genomes of four *Rhodococcus* species, including *R. erythropolis*, harbor a considerably greater number of biosynthetic gene clusters compared to other actinobacteria, which are themselves recognized as rich reservoirs of natural products (Doroghazi and Metcalf, 2013). Specifically, in the biocontrol agent *R. erythropolis* R138 strain, genomic analyses identified nearly 90 biosynthetic gene clusters, thereby revealing the genetic potential for the biosynthesis of a highly diverse array of natural products (Ceniceros et al., 2017b; Kwasiborski et al., 2015). Investigating the regulatory mechanisms of these metabolic pathways could enable the discovery of novel antimicrobial molecules by unlocking otherwise silent pathways. Indeed, most of these pathways are not expressed efficiently or not at all under routine laboratory culturing conditions. The regulation of these intricate specialized metabolic pathways by specific signaling molecules has been well established in the model organism *S. coelicolor*, the QS system of which requires ScbA for GBL signal production and ScbR for signal detection (Takano, 2006).

### 4.1 Emission of GBL signals by *Rhodococcus erythropolis*

Analysis of the genomes of *Rhodococcus* type strains showed that *scbA* and *scbR* homologs are broadly distributed across the genus, underscoring the importance of the GBL-dependent QS system in rhodococci. Based on its conserved occurrence across all sequenced *R. erythropolis* strains, we propose an extended GBL gene cluster comprising three additional genes encoding an epimerase, a FAD-containing monooxygenase, and a transcriptional regulator. The GBL biosynthesis pathway in *Streptomyces* remains partially elucidated. ScbA, an acyltransferase, catalyzes the initial step involving the condensation of dihydroxyacetone phosphate with a *β*-ketoacid to generate a fatty acid ester intermediate, which is converted into a GBL molecule by three steps of dephosphorylation, aldol condensation, and reduction as previously proposed (Ceniceros et al., 2017a; Kato et al., 2007; Sakuda et al., 1992). Homologs of the epimerase-encoding gene have been studied in *Streptomyces venezuelae* and *S. virginiae*, designated *jadW2* (Wang and Vining, 2003) and *barS2*, respectively, with experimental evidence indicating that *barS2* is essential for GBL synthesis (Lee et al., 2008). In the GBL biosynthetic pathway proposed by Niu et al. (2016), the epimerase enzyme likely participates in the dehydration step, which occurs before the NAD-dependent reduction that generates GBL molecules. Monooxygenase encoding gene was detected in the near vicinity of *scbA* gene in *S. lavendulae* and *S. virginiae* genomes and has been demonstrated in *Streptomyces rochei* and *Streptomyces avermitilis* to participate in the biosynthesis of butenolides, other autoregulators in *Streptomyces*, that are structurally related to GBLs differing by the presence of an unsaturated lactone ring (Kitani et al., 2011; Teshima et al., 2020). An obvious synteny with the GBL gene clusters of *Rhodococcus* strains was observed in contrast to those of the *Streptomyces* strains which were poorly conserved, as previously reported (Biarnes-Carrera et al., 2015). This GBL gene cluster organization reflects the homogeneous nature of GBL production in *Rhodococcus* species, limited so far to *R. jostii*, *R. rhodnii* and *R. rhodochrous*, all producing exclusively A-factor-type GBLs (Kudo et al., 2025) and contrasts with the molecular diversity observed in *Streptomyces* strains, which synthesize all three GBL types: A-factor-, IM-2-, and VB-type GBLs (Creamer et al., 2021; Kudo et al., 2025). In the *R. erythropolis* species, GBL production was investigated by LC–MS and by bioassay using a GBL-specific reporter strain. The *R. erythropolis* R138 wild-type strain produced GBL(−like) molecules, that may correspond to A-Factor-type, in contrast to the R138 Δ*scbA* mutant, which did not produce them. This production was formerly examined in the type strain *R. erythropolis* NBRC 15567^T^, where no GBL was detected, likely due to a partial deletion of the *afsA* conserved repeat domains in its *scbA* homolog (Kudo et al., 2025). No such deletion was observed in the *scbA* homolog of the study strain *R. erythropolis* R138 nor in other *R. erythropolis* strains, indicating that these strains may also produce GBL molecules.

### 4.2 Reception of GBL signals in *Rhodococcus erythropolis*

The detection of GBL molecules is mediated by ScbR homolog proteins (Takano, 2006). These GBL receptors belong to the TetR transcriptional regulator family, which represents one of the largest and most studied groups of one-component systems. They usually act as transcriptional repressors by binding to the promoter of target genes, and generally function as autoregulators controlling their own gene expression (Cuthbertson and Nodwell, 2013). In the model species *S. coelicolor*, ScbR not only represses the transcription of its own gene but also controls *scbA* expression in a growth-phase-dependent manner (Takano et al., 2001; Biarnes-Carrera et al., 2015). In addition, ScbR regulates the secondary metabolism gene clusters involved in the production of the three antibiotics coelimycin (Takano et al., 2005), actinorhodin, and undecylprodigiosin (Takano et al., 2000; Takano et al., 2001). Thus, the GBL-based QS system in *S. coelicolor* is tightly regulated at the transcriptional level in order to coordinate the onset of antibiotic production, and avoid the toxicity of the end product for producing strains. Such a regulatory system was investigated in the study strain *R. erythropolis* R138. The 3D structures of ScbR from *S. coelicolor* and *R. erythropolis* R138 were modeled based on the crystal structures of CprB from *S. coelicolor* (Bhukya et al., 2014; Natsume et al., 2004) and TylP from *S. fradiae* (Mohd-Sharif et al., 2017), both of which are pseudo-GBL receptors, the binding of which to GBLs has not yet been demonstrated. To date, attempts to crystallize genuine GBL receptors confirmed to bind GBLs have been unsuccessful (Biarnes-Carrera et al., 2015). Structural superposition of the two 3D models revealed a high degree of conservation between the two ScbR proteins from *S. coelicolor* and *R. erythropolis* R138, implying a similar functional role. GBL recognition is mediated by the C-terminal region of ScbR (Bhukya et al., 2014). GBL molecules share a common lactone ring of 3R-hydroxymethyl-1,4-butyrolactone and a C_2_ *n*-alkane residue, which is specific for each species and has a unique stereochemistry, chain length or branching (Takano, 2006). Biswas et al. (2014) demonstrated that the common butyrolactone ring directly interacts with the binding pocket of the receptor by exploiting the presence of a conserved tryptophan residue (Trp-127) in the cavity to monitor its fluorescence quenching upon the addition of chemically synthesized GBLs. This result indicates that this pocket is responsible for the recognition of the butyrolactone ring, whereas the *n*-alkane C_2_ residue may be responsible for the specificity of the binding. Notably, ScbR receptors have been reported to exhibit high specificity and sensitivity for their cognate GBLs, in contrast to AHL-based QS systems, which are described as promiscuous (Biarnes-Carrera et al., 2015; Takano, 2006).

To investigate the role of ScbR in *scbR* and *scbA* expression regulation, *gfp*-based transcriptional fusions were constructed and analyzed by dot blot and CLSM. In the pEPR1 promoter-probe vector, insertion of the *scbR* promoter upstream of the *gfp* reporter gene resulted in detectable GFP production, whereas insertion of the *scbA* promoter did not. In the presence of ScbR, no activity was detected from the *scbR* promoter, in contrast to the *scbA* promoter, suggesting that ScbR acts as a repressor of *scbR* transcription and as an activator of *scbA* expression. A similar regulatory pattern was observed in *S. coelicolor*, where ScbR represses its own transcription while inducing *scbA* expression, likely in cooperation with additional factors only during the transition from exponential to stationary growth phase (Takano et al., 2001; Martin Sanchez, 2016). Such a dual regulatory role of the ScbR homolog JadR3 was also reported in *S. venezuelae*. JadR3 promotes jadomycin biosynthesis by activating transcription of the transcriptional activator encoding gene *jadR1*, while repressing its own gene as well as the transcriptional repressor encoding gene *jadR2* and the GBL synthase encoding genes *jadW1, jadW2*, and *jadW3* (Zou et al., 2014). In the study of *R. erythropolis* R138 strain, the regulation of *scbA* expression appeared to be more complex, involving an additional transcriptional regulator, the gene of which is located upstream of *scbA* and belongs to the LuxR family. Through transcriptional fusion analyses, we showed that the *scbA* promoter exhibited activity in the presence of the LuxR-like regulator, suggesting a role of this factor as an activator of the *scbA* gene expression. This activator function is described for most LuxR-like proteins that activate the expression of *luxI* homolog genes involved in the production of the Gram-negative bacteria QS AHLs (Cai and Zhang, 2024). However, this LuxR homolog lacks a linked LuxI synthase and functions as an orphan or “solo” LuxR, playing a crucial role in bacterial signaling for both interbacterial and host–bacterial interactions (Venturi and Fuqua, 2013). In *Rhodococcus* sp. strain p52, a LuxR solo has been demonstrated to regulate recalcitrant aromatic compound biodegradation (Fu et al., 2025). Based on genome analysis of rhodococcal GBL clusters, the LuxR-encoding gene appears broadly conserved across *Rhodococcus* type strains, except in those clusters where *scbA* and *scbR* overlap at their 3′ ends. This convergent and overlapping arrangement of *scbA* and *scbR* in some *Rhodococcus* species suggests that the expression of one member of a gene pair might be antagonized by the expression of the other member, either via RNA polymerase collisions or by hybridization of the two complementary mRNAs. This gene arrangement forms a regulatory system controlling gene expression (Tsai and Winans, 2010). These observations suggest that *scbA* expression may be governed by two distinct regulatory mechanisms: one mediated by the LuxR-like transcriptional regulator, and the other arising from the *scbA*-*scbR* convergent genomic topology, with a 3′ end overlap. A similar genomic topology is found in the *luxR*-*luxI* homologous QS systems of several Gram-negative bacteria (Tsai and Winans, 2010). To our knowledge, this is the first report supporting a role for a LuxR regulator in the regulation of a QS system in Gram-positive bacteria.

### 4.3 Interspecies crosstalk via ScbA/ScbR QS systems

ScbA/ScbR homolog systems function as QS systems that rely on the production and detection of diffusible GBL signals to regulate secondary metabolism in *Streptomyces* (Biarnes-Carrera et al., 2015). The role of ScbA was investigated in *R. erythropolis* and *S. coelicolor* interactions. The ScbA protein structure of *R. erythropolis*, modeled using AlphaFold, displayed strong structural similarity to that of *S. coelicolor*, and the key amino acid residues required for GBL biosynthesis were conserved, suggesting a similar enzymatic function. In *Streptomyces*, GBLs produced by ScbA are known to induce sporulation (Takano, 2006). When incubated in proximity on agar plates, the *R. erythropolis* R138 wild-type strain triggered spore formation in the *S. coelicolor* LW94 mutant strain lacking *scbA*, whereas the R138 Δ*scbA* mutant failed to do so. Furthermore, attraction of *S. coelicolor* toward *R. erythropolis* R138 was observed under conditions in which the latter produced GBL(−like) molecules. To our knowledge, this phenomenon has not been reported previously. These results indicated that these two strains were able to communicate and that *scbA*-dependent compounds secreted by *R. erythropolis* affected morphological differentiation in the GBL-deficient *S. coelicolor* strain. These compounds may correspond to GBL homolog molecules produced by *R. erythropolis* R138 and detected by *S. coelicolor* LW94. These GBL signals were first described in *S. griseus* to control cellular differentiation and secondary metabolism (Horinouchi and Beppu, 2007). *R. erythropolis* R138 produces GBL(−like) molecules that may belong to the A-Factor-type. This identification is not fully consistent with the ScbA phylogenetic analysis, which showed a closer relationship to the ScbA homolog of *S. virginiae,* reported to produce VB-type GBLs (Creamer et al., 2021). According to Hirata et al. (2025), the type of signaling molecules cannot be reliably predicted based on the sequence homology of the corresponding biosynthetic enzymes. For instance, despite sharing only 45% sequence identity, the ScbA proteins from *S. coelicolor* and *S. venezuelae* are capable of producing the same GBL molecule. As observed in *S. griseus*, *R. erythropolis* R138 appears unable to produce ketoreductases such as ScbB from *S. coelicolor*, which are required to reduce the ketone group to a hydroxyl group and thereby generate IM-2- and VB-type GBLs.

Further investigations are required to identify genes whose expression is controlled by the ScbA/ScbR system in the genus *Rhodococcus*. Comparative transcriptomic or proteomic analyses between the *R. erythropolis* R138 wild-type strain and an *scbR*-deletion mutant strain may uncover metabolic pathways that are tightly controlled by the GBL-based QS system. Interestingly, members of the *R. erythropolis* species are known to carry various metabolic pathways dealing with lactones (Afriat et al., 2006; Barbey et al., 2012; Moreno-Horn et al., 2016; Cirou et al., 2007). Among them, the Qsd (Quorum-sensing signal degradation) pathway of *R. erythropolis* R138 strain is able to disrupt AHL-communication in various Gram-negative bacteria (Barbey et al., 2018; Bourigault et al., 2021). This quorum-quenching activity mainly relies on the hydrolysis of the lactone ester bond of numerous AHLs by the low-specificity lactonase QsdA. Because GBLs share a similar core-structure to AHL signals, a *γ*-butyrolactone ring, we hypothesize that the Qsd pathway of *R. erythropolis* R138 could also modulate its own GBL-based QS communication. The latter QS system exhibits another similarity to AHL-based QS systems, specifically the role of the LuxR protein in regulating the production of QS signals. This observation further supports the potential cross-talk between GBL and AHL signaling systems proposed recently by Liu et al. (2021).

## Data Availability

The datasets presented in this study can be found in online repositories. The names of the repository/repositories and accession number(s) can be found in the article/Supplementary material.

## Footnotes

1
https://www.ncbi.nlm.nih.gov/datasets/genome/

2
https://itol.embl.de/

## Supplementary material

SUPPLEMENTARY FIGURE S1 Amino acid sequence alignment and AfsA repeat conserved functional domains in ScbA from *R. erythropolis* R138 and *S. coelicolor.* The amino acid sequences were aligned using ClustalW aligorithm. The two red boxes indicate the two functional domains corresponding to the AfsA repeats. Asterisks indicate fully conserved residues in all sequences. ‘:’ (colon) symbol means conserved substitutions. ‘.’ (dot) symbol indicates partially conserved substitutions. Amino acid residues essential for the enzyme activity (Hsiao et al., 2009; Martin Sanchez, 2016) are shown with numbers and highlighted with red asterisks.

SUPPLEMENTARY FIGURE S2 Amino acid sequence alignment and deduction of functional domains in ScbR from *R. erythropolis* R138 and *S. coelicolor.* The amino acid sequences were aligned using the ClustalW aligorithm. Functional domain and residue assignments are inferred from *S. coelicolor* CprB and *S. fradiae* TylP, the only crystallized ScbR homologs reported to date (Natsume et al., 2004; Mohd-Sharif et al., 2017). Residues forming *α*-helices are boxes and labeled. Dashed box marks the DNA binding domain (α1-3) with α2 and α3 forming a helix-turn-helix motif. Bold box indicates the regulatory domain (α5-10). α-Helices 4–8, involved in forming the γ-butyrolactone binding pocket, are indicated by shaded boxes. Asterisks indicate fully conserved residues in all sequences. ‘:’ (colon) symbol means conserved substitutions. ‘.’ (dot) symbol indicates partially conserved substitutions. Amino acid residues essential for DNA binding (Pro and Val; Onaka and Horinouchi, 1997; Sugiyama et al., 1998) and ligand binding (Trp; Sugiyama et al., 1998) are highlighted with red asterisks.

SUPPLEMENTARY FIGURE S3 Mass spectra of the fragmentation (NCE 40) of the ion precursors m/z 241.145 **(A)** and m/z 255.160 **(B)** at 15.07–15.80 and 15.57–16.30 retention times, respectively, from the LC–MS/MS analysis of ethyl acetate extracts of the *R. erythropolis* R138 wild-type, Δ*scbA* and Δ*scbA*-*scbA* strains. A) MS/MS spectra corresponding to the chromatographic peaks eluting at 15.07 and 15.80 min for m/z 241.145, shown in the left and right panels, respectively. B) MS/MS spectra corresponding to the chromatographic peaks eluting at 15.60 and 16.30 min for m/z 255.160, shown in the left and right panels, respectively.

SUPPLEMENTARY FIGURE S4 Detection of GBL molecules in the *R. erythropolis* R138 ethyl acetate culture extracts by a GBL-specific reporter assay. In the absence of kanamycin (−km), the *S. coelicolor* reporter strain grew over the entire surface of the agar plates (*S. coelicolor* growth control). In the presence of kanamycin (+km), the reporter strain formed a halo of growth (purple) around the sample application site at the center of the agar plates only when GBL molecules were present in the sample. The samples tested are the ethyl acetate extracts of *R. erythropolis* R138, and of *S. coelicolor* M145, which was used as a positive control. The negative control consisted of the solvent used to resuspend the ethyl acetate extracts prior to application at the center of the agar plates. The pictures shown are representative of three independent experiments.

SUPPLEMENTARY FIGURE S5 Analysis of *scbA* and *scbR* promoter activity through measurement of GFP production in *R. erythropolis* R138 strains carrying transcriptional fusions with the *gfp* reporter gene. Dot blot assay results are presented as histograms showing the spot intensity obtained with anti-GFP antibodies normalized to the spot intensity obtained with anti-RecA antibodies. The dashed line indicates the signal threshold, defined as the GFP/RecA intensity ratio measured for the *R. erythropolis* R138 strain with the promoterless pEPR1 plasmid. Significant differences (Mann and Whitney test; *p*-value < 0.05) in GFP production are indicated with an asterisk.

SUPPLEMENTARY VIDEO 1 Overlay of the predicted 3D structures of ScbA to compare the proteins from *R. erythropolis* R138 and *S. coelicolor.* Alphafold was used to predict the 3D structure of ScbA from *R. erythropolis* R138 (dark color) and *S. coelicolor* (light color). The two central α helices form part of the two conserved AfsA domains (Pfam form the03756) that contain the putative active site of ScbA. The conserved amino acid residues E78, R81, E240, R243 in the putative ScbA active site and R174 in the loop region connecting the AfsA repeats which were identified as essential for enzyme activity (Hsiao et al., 2007; Martin Sanchez, 2016) are depicted in red. Protein structures were visualized by Pymol.

SUPPLEMENTARY VIDEO 2 Overlay of the predicted 3D structures of ScbR to compare the proteins from *R. erythropolis* R138 and *S. coelicolor.* Alphafold was used to predict the 3D structure of ScbR from *R. erythropolis* R138 (dark color) and *S. coelicolor* (light color). The transcriptional factor ScbR consists of a helix-turn-helix DNA-binding motif in the N-terminal region and a regulatory ligand-binding domain in the C-terminal region. Protein structures were visualized by Pymol.
