# Supplementary material for: Characterization of a quorum-sensing communication based on gamma-butyrolactones in Rhodococcus erythropolis
Source: Front Microbiol. 2026 Jul 27;17:1849643. doi: 10.3389/fmicb.2026.1849643 (PMC13454427; doi:10.3389/fmicb.2026.1849643)
Supplement: Supplementary file 1 [file Table_1.docx]

**Supplementary Material**

**TABLE S1. Bacterial strains and plasmids**

| **Strain or plasmid** | **Relevant characteristic(s)** | **Source or reference** |
| --- | --- | --- |
| ***Escherichia coli*** |  |  |
| S17-1 | recA pro hsdR RP4-2-Tc::Mu-Km::Tn*7* | (Simon et al., 1983)^[1]^ |
| Top10^®^ | F- mcrA Δ(mrr-hsdRMS-mcrBC) Φ80lacZΔM15 ΔlacX74 recA1 araD139 Δ(araleu)7697 galU galK rpsL (StrR) endA1 nupG | Lab collection |
| ***Rhodococcus erythropolis*** | | |
| R138 | AHL-degrading isolate obtained from hydroponic culture of potato plants | (Cirou et al., 2007)^[2]^ |
| R138 Δ*scbA* | R138 with a 831 bp fragment deleted from the *scbA* gene | This study |
| R138 Δs*cbA-scbA* | R138 Δ*scbA* strain in which the wild-type *scbA* gene was integrated into the chromosome at the origin locus | This study |
| R138 pEPR1 | R138 transformed by pEPR1 empty vector with a promoterless *gfp* gene; Km^R^ | This study |
| R138 pEPR1-P*scbA::gfp* | R138 transformed by pEPR1 containing a transcriptional fusion of the promoter of *scbA* gene and a GFP-encoding gene (P*scbA*::*gfp*); Km^R^ | This study |
| R138 pEPR1-*luxR*- P*scbA::gfp* | R138 transformed by pEPR1-P*scbA*::*gfp* containing an extended promoter region of *scbA* including *luxR*; Km^R^ | This study |
| R138 pEPR1- P*scbA::gfp*-P_45_*::luxR* | R138 transformed by pEPR1-P*scbA*::*gfp* containing a transcriptional fusion of the strong constitutive *Corynebacterium glutamicum* P_45_ promoter and R138 *luxR* gene (P_45_*::luxR*); Km^R^ | This study |
| R138 pEPR1-P*scbA::gfp* -P_45_*::scbR* | R138 transformed by pEPR1-P*scbA*::*gfp* containing a transcriptional fusion of the P_45_ promoter and *scbR* gene (P_45_*::scbR*); Km^R^ | This study |
| R138 pEPR1-P*scbA::gfp* -P_45_*::scbR-*P_45_*::luxR* | R138 transformed by pEPR1-P*scbA*::*gfp* containing a transcriptional fusion of the P_45_ promoter and *scbR* gene (P_45_*::scbR*) and a transcriptional fusion of the P_45_ promoter and *luxR* gene (P_45_*::scbR*); Km^R^ | This study |
| R138 pEPR1-P*scbR::gfp* | R138 transformed by pEPR1 containing a transcriptional fusion of the *scbR* gene promoter and a GFP-encoding gene; Km^R^ | This study |
| R138 pEPR1-P*scbR::gfp* -P_45_*::scbR* | R138 transformed by pEPR1-P*scbR*::*gfp* containing a transcriptional fusion of the P_45_ promoter and *scbR* gene (P_45_*::scbR*); Km^R^ | This study |
| ***Streptomyces coelicolor*** |  |  |
| M145 | Wild type strain of *S. coelicolor* | (Bentley et al., 2002) |
| LW94/pTE134 | A *S. coelicolor* M145 deletion mutant of *scbA* and *scbR* genes, transformed with the pTE134 plasmid containing the *scbR* gene and a kanamycin resistance gene under the control of a γ-butyrolactone inducible promoter; GBL reporter strain | (Biarnes-Carrera et al., 2018b; Hsiao et al., 2009) |
| **Plasmids** |  |  |
| pAKE604 | Conjugative suicide vector in *R. erythropolis* R138; Replicative vector in *E. coli*; Km^R^ | (El-Sayed et al., 2001) |
| pAKE604 Δ*scbA* | pAKE604 containing the *scbA* upstream and downstream regions; Km^R^ | This study |
| pAKE604 *scbA* | pAKE604 containing the wild type *scbA* gene and its upstream and downstream regions; Km^R^ | This study |
| pEPR1 | Vector for testing *in vivo* promoter activities in *R. erythropolis*; Km^R^ | (Knoppová et al., 2007) |
| pEPR1-P*scbA::gfp* | pEPR1 vector containing a transcriptional fusion with the *gfp* gene under the control of the *scbA* promoter; Km^R^ | This study |
| pEPR1-*luxR*- P*scbA::gfp* | pEPR1 vector containing a transcriptional fusion with the *gfp* gene under the control of an extended promoter region of *scbA* including *luxR*; Km^R^ | This study |
| pEPR1-P*scbA::gfp*-P_45_*::luxR* | pEPR1 vector containing a transcriptional fusion with the *gfp* gene under the control of the *scbA* gene promoter and the transcriptional P_45_*::luxR* under the control of the P_45_ promoter; Km^R^ | This study |
| pEPR1-P*scbA::gfp*-P_45_*::scbR* | pEPR1 vector containing a transcriptional fusion with the *gfp* gene under the control of the *scbA* gene promoter and the transcriptional P_45_*::scbR* under the control of the P_45_ promoter; Km^R^ | This study |
| pEPR1-P*scbA::gfp*-P_45_*::scbR-* P_45_*::luxR* | pEPR1 vector containing a transcriptional fusion with the *gfp* gene under the control of the *scbA* gene promoter and the transcriptional P_45_*::scbR* and P_45_*::luxR* fusions under the control of the P_45_ promoter; Km^R^ | This study |
| pEPR1-P*scbR::gfp* | pEPR1 vector containing a transcriptional fusion with the *gfp* gene under the control of the *scbR* promoter; Km^R^ | This study |
| pEPR1-P*scbR::gfp* -P_45_*::scbR* | pEPR1 vector containing a transcriptional fusion with the *gfp* gene under the control of the *scbR* promoter and the transcriptional P_45_*::scbR* fusion under the control of the P_45_ promoter; Km^R^ | This study |
| pRAG5 | Plasmid containing the strong constitutive *C. glutamicum* promoter P_45_ | (Knoppová et al., 2007) |

Km^R^ indicates resistance to kanamycin.

1. Simon, R., Priefer, U., and Pühler, A. (1983). A broad host range mobilization system for in vivo genetic engineering: transposon mutagenesis in gram negative bacteria. Biotechnology 1, 784–791.
