# Supplementary material for: Characterization of a quorum-sensing communication based on gamma-butyrolactones in Rhodococcus erythropolis
Source: Front Microbiol. 2026 Jul 27;17:1849643. doi: 10.3389/fmicb.2026.1849643 (PMC13454427; doi:10.3389/fmicb.2026.1849643)
Supplement: Supplementary file 2 [file Table_2.docx]

**Supplementary Material**

**TABLE S2. Primers used for mutagenesis and transcriptional fusion constructions**

| **Primer name** | **Sequence*** | **Reference** |
| --- | --- | --- |
| ***scbA* deletion** |  |  |
| scbA_up_fw | 5’ TAATAA*TCTAGA*CTATCGCTGTCACCCTTCGT 3’ | This study |
| scbA_up_rv | 5’ TAATAA*AAGCTT*ACTGTGCGTGTGTATTCCAT3’ | This study |
| scbA_do_fw | 5’ TAATAA*AAGCTT*CGCGGAACTTGAACTTACAG 3’ | This study |
| scbA_do_rv | 5’ TAATAA*GGATCC*TACGACACGCAGCAAATGAC 3’ | This study |
| **Revertant strain construction** | | |
| scbA_up_fw | 5’ TAATAA*TCTAGA*CTATCGCTGTCACCCTTCGT 3’ | This study |
| scbA_do_rv | 5’ TAATAA*GGATCC*TACGACACGCAGCAAATGAC 3’ | This study |
| **Transcriptional fusion constructions** | |  |
| PscbA_fw | 5’ TAATAA*TCTAGA*TTCTCTACGGCGGTTCGAAT 3’ | This study |
| PscbA_rv | 5’ TAATAA*GGATCC*TGATTGAATGCACCTTTCGA 3’ | This study |
| luxRPscbA_fw | 5’ TAATAA*TCTAGA*AGAACATTGAGAGTGCGACG 3’ | This study |
| PscbR_fw | 5’ TAATAA*TCTAGA*GTGGACGCCTGGCTCATA 3’ | This study |
| PscbR_rv | 5’ TAATAA*GGATCC*GATCTACCCAGCTCTCGTTG 3’ | This study |
| P45_fw | 5’ TAATAA*ATGCAT*AATACACTGCCCGTTTCAGG 3’ | This study |
| P45_rv | 5’ TAATAA*CCATGG*TGGCGACCAGCTCTTG 3’ | This study |
| scbR_fw | 5’ TAATAA*AAGCTT*ATGCAGGAGCGTGCGGTC 3’ | This study |
| scbR_rv | 5’ TAATAA*GCTAGC*TTATCAACGGCGACGGCGACGACG  AGCGCTCGGTGCTTCCCACT 3’ | This study |
| luxR_fw | 5’ TAATAA*CCATGG*ATGACACACTCCAGCACAGT 3’ | This study |
| luxR_rv | 5’ TAATAA*ATGCAT*TTATCAACGGCGACGGCGACGAC  GGAAGCGTCCGATCCGAAGTT 3’ | This study |
|  |  |  |

*All primers used in this study were synthesized by Eurogentec and are based on *R. erythropolis* R138 genome sequence (NCBI accession no. NZ_CM002793.1); Restriction sites are shown in italics.
