# Supplementary material for: Characterization of a quorum-sensing communication based on gamma-butyrolactones in Rhodococcus erythropolis
Source: Front Microbiol. 2026 Jul 27;17:1849643. doi: 10.3389/fmicb.2026.1849643 (PMC13454427; doi:10.3389/fmicb.2026.1849643)
Supplement: Supplementary file 3 [file Table_3.docx]

**Supplementary Material**

**TABLE S3. Genomic environment of the GBL gene cluster in *Rhodococcus erythropolis* species**

| ***R. erythropolis* species** | **Genomic environment of the GBL cluster** |
| --- | --- |
| - BG43  - FRAMRAS EGC1617 bin 27  - Fur100  - IEGM1309  - IEGM453  - IEGM487  - Jub83  - MI2  - NPDC096763  - NPDC015230  - NPDC015711  - NPDC18573  - NPDC018581  - NPDC018918  - NPDC097249  - NPDC097508  - NPDC097687  - NPDC097772  - NPDC097108  - NPDC97886  - NPDC097878  - NPDC097874  - NPDC097847  - NPDC097803  - NPDC058306  - PR4  *-* R138  - R85  - 2NaKV04  - 164 chir2E |  |
| - CGMCC1 2362  - NBRC 15567 |  |
| - NI 86 21 |  |
| -PFA D8 1 |  |
| - L E1 T20 A bin57 |  |
| - HX7  - IEGM1189  - ISL71  - L E1 T20 Bin57  - myb53  - NPDC05 0034  - NPDC056490  - NPDC092078  - S2W2  - XP |  |
| - SK121 |  |
| - IEGM91 |  |
| - JCM2895 |  |
| - NPDC005162  - T1801  - VSD3 |  |
| - CCM2595  - NCTC8036  - NSx2 |  |
| - D310 1 |  |
| - ATCC 15903  - IEGM1399  - NRRLB16532 |  |
| - NPDC058281 |  |
| - Ac1659  - ACN1  - BIGb0270  - cas922i  - CERE8  - KB1  - X5 |  |
| - IEGM1415 |  |
